# Genetic analysis and QTL mapping for pericarp thickness in maize (*Zea mays* L.)

**DOI:** 10.1186/s12870-024-05052-1

**Published:** 2024-04-25

**Authors:** Guantong Gong, Haitao Jia, Yunqi Tang, Hu Pei, Lihong Zhai, Jun Huang

**Affiliations:** 1 Guangdong Provincial Key Laboratory of Plant Molecular Breeding, South China Agricultural University, Guangzhou, 510642 China; 2 Hubei Key Laboratory of Food Crop Germplasm and Genetic Improvement, Food Crops Institute, Hubei Academy of Agricultural Sciences, Wuhan, 430064 China; 3 Basic School of Medicine, Hubei University of Arts and Science, Xiangyang, 441053 China

**Keywords:** Maize, Pericarp thickness, SNP, QTL mapping

## Abstract

Proper pericarp thickness protects the maize kernel against pests and diseases, moreover, thinner pericarp improves the eating quality in fresh corn. In this study, we aimed to investigate the dynamic changes in maize pericarp during kernel development and identified the major quantitative trait loci (QTLs) for maize pericarp thickness. It was observed that maize pericarp thickness first increased and then decreased. During the growth and formation stages, the pericarp thickness gradually increased and reached the maximum, after which it gradually decreased and reached the minimum during maturity. To identify the QTLs for pericarp thickness, a BC_4_F_4_ population was constructed using maize inbred lines B73 (recurrent parent with thick pericarp) and Baimaya (donor parent with thin pericarp). In addition, a high-density genetic map was constructed using maize 10 K SNP microarray. A total of 17 QTLs related to pericarp thickness were identified in combination with the phenotypic data. The results revealed that the heritability of the thickness of upper germinal side of pericarp (UG) was 0.63. The major QTL controlling UG was *qPT1-1*, which was located on chromosome 1 (212,215,145–212,948,882). The heritability of the thickness of upper abgerminal side of pericarp (UA) was 0.70. The major QTL controlling UA was *qPT2-1*, which was located on chromosome 2 (2,550,197–14,732,993). In addition, a combination of functional annotation, DNA sequencing analysis and quantitative real-time PCR (qPCR) screened two candidate genes, *Zm00001d001964* and *Zm00001d002283*, that could potentially control maize pericarp thickness. This study provides valuable insights into the improvement of maize pericarp thickness during breeding.

**Supplementary Information:**

The online version contains supplementary material available at 10.1186/s12870-024-05052-1.

## Introduction

Pericarp is the outer protective tissue of maize kernel. It protects the embryo and endosperm from mechanical damage or pests. Moreover, it regulates the rate of dehydration and maturation of the kernel and water absorption for germination [1, 2]. Maize pericarp thickness is reported to be associated with seed quality [3], popcorn quality [4], resistance to pathogens [5], seed maturity, and moisture content [6]. Furthermore, pericarp thickness is a quality-related factor for sweet corn breeding. Thin pericarp is a desirable trait for sweet corn because it improves the tenderness [7]. Pericarp thickness negatively correlates with tenderness and influences the resistance to mastication [8]. Therefore, the selection of appropriately thin pericarp is crucial for the quality breeding of maize.

The development process of maize pericarp is divided into four stages: undeveloped, thickening, expansion, and strengthening using the hierarchical clustering and PCA analysis of high temporal resolution transcriptome [9]. Then, it was found that from 15 to 23 day after pollination (DAP), the pericarp thickness of M03 and M08 showed a decreasing trend, and then gradually stabilized from the dynamic measurements of pericarp thickness with these two lines [10]. Certainly, the differences in pericarp thickness of various maize materials were also investigated. The pericarp thickness of 19 maize materials (genotypes *Su*, *su*, *sh2*, and *suse*) was analyzed and showed that *SuSu* had the thickest pericarp and *suse* had the thinnest pericarp [11]. The molecular mechanisms of genes that control pericarp thickness vary. *pericarp color 1* gene increases maize pericarp thickness through the accumulation of phlobaphene [12]. And silencing the *SlPRE2* gene affected the plant's response to gibberellins resulting in reduced tomato fruit size and pericarp thickness [13]. Gan et al. found that the reduction in tomato pericarp thickness was mainly due to a reduction in cell number, which led to a reduction in cell division. In addition, endogenous cytokinins were demonstrated to regulate pericarp cell division and fruit size using transcriptome analysis [14].

Quantitative trait loci (QTL) mapping is a key step in exploring the genetic mechanisms of the complex quantitative trait of maize pericarp thickness. The genes controlling maize pericarp thickness ranged from 1.4 to 5.9, with an average narrow-sense heritability of 55.2%, and pericarp thickness is a highly heritable trait that is not strongly influenced by the environment [15, 16]. Helm et al. classified the pericarp into six positions: upper, middle and lower part of the germinal and non-germinal surfaces. And they found that the narrow-sense heritability of pericarp thickness was estimated to be up to 80% [17]. Choe et al*.* measured pericarp thickness at five different positions on maize and reported that the thickness at all five different positions exhibited high heritability and correlation and most QTLs were linked to more than one pericarp thickness position. This suggested that the pericarp thickness at different positions on maize is controlled by common genes with multiple effects [16]. Wang et al*.* used different molecular markers to investigate the QTLs that control pericarp thickness in maize and reported that three genes were associated with pericarp thickness on chromosomes 1, 2, and 6 [18]. Park et al. found four QTLs associated with pericarp thickness, which were located on chromosomes 4, 5, 8, and 9 [19].

In this study, we first investigated the dynamics of maize pericarp thickness during kernel development in normal corn, sweet corn, and waxy corn inbred lines. In addition, we constructed the BC_4_F_4_ population using two maize inbred lines with different pericarp thicknesses [B73 (thick pericarp) and Baimaya (thin pericarp)] and identified the QTLs for pericarp thickness based on inclusive composite interval mapping. The results of our study may provide a theoretical basis for changing the thickness of maize pericarp during maize breeding.

## Materials and methods

### Plant materials and field experiments

The materials used in this study was provided by the maize group of South China Agricultural University. To investigate the dynamic changes of maize pericarp thickness, sweet corn inbred line ZF1, waxy corn inbred line TN113, and normal corn inbred line N75 were used as materials. They were planted in Zengcheng District, Guangzhou City, Guangdong Province, China (ZC, approximately 113°E and 23°N) in August 2022. In terms of QTL mapping for pericarp thickness, two parentlines B73 and Baimaya were selected to construct the BC_4_F_4_ population. B73 (thick pericarp) was used as a recurrent parent, and Baimaya (thin pericarp) and was choosed as donor parent. In February 2022, 318 BC_4_F_4_ families and their parents were planted in Huanggang City, Hubei Province, China (HG, approximately 115°E and 30°N), and Gucheng County, Xiangyang City, Hubei Province, China (GC, approximately 111°E and 31°N). The 318 BC_4_F_4_ lines and their parents were also planted in August 2022 in Zengcheng District, Guangzhou City, Guangdong Province, China (approximately 113°E and 23°N). In total, 10 plants of each family line or parent were grown with 2 rows. The length of the rows was 3 m. The distance between rows was 70 cm, and the distance between plants was 25 cm. The ears were covered with sulfuric acid paper bags to ensure self-fertilization before the silking stage. Locally recommended herbicide and pesticide programs were followed for crop management.

### Pericarp collection and phenotypic analysis

To monitor the changes in maize pericarp, the successfully fertilized kernels of ZF1, TN113, and N75 were collected every 2 days from the 5th day after self-pollination. For QTL mapping, the ears from the BC_4_F_4_ population and parents were collected at 21 DAP. Three self-pollinated ears were sampled from each genotype and stored at -20°C.

Ten kernels were collected from the middle of each ear from the above three self-pollinated ears. One-third of the head of the corn kernel was transversely cut off with a double-edged razor blade, followed by slicing the samples with a thickness of < 1 mm on a slide and pipetting 30 μL Safranin O solution (Diluted to 0.05 g/L. Roles-Bio, Guangzhou, China) into the center of the samples. After staining for 10 to 15 s, excess staining solution was removed using a filter paper, and the stained sections were observed under a Zeiss light microscope (Zeiss, Shanghai, China). The thickness of upper germinal side and upper abgerminal side of pericarp and the number of pericarp cell layers were recorded. Dividing pericarp thickness by the number of cell layers as a reference value for individual pericarp cell thickness. The observed values of pericarp thickness and the number of pericarp cell layers for each genotype were determined as the average values obtained from three ears, and these averaged values were utilized for subsequent analysis.

IBM SPSS Statistics 26 software (IBM Corp. Released 2019; IBM SPSS Statistics for Windows, Version 26.0. Armonk, NY: IBM Corp) was used to perform descriptive statistics, normal distribution plotting, normality tests (P-P plots), and Pearson’s correlation analysis [20, 21]. The broad-sense heritability (${h}_{b}^{2}$) was estimated as ${h}_{b}^{2}={V}_{ g}^{2}/\left({V}_{ g}^{2}+{V}_{ ge}^{2}/e+{V}_{ e}^{2}/re\right)$. ${V}_{ g}^{2}$ is the genetic variance. ${V}_{ e}^{2}$ is the residual variance. ${V}_{ ge}^{2}$ is the genotype-by-environment interaction. e represents the number of environments and r represents the number of replicates in each environment [22].

### Construction of linkage maps and QTL mapping for pericarp thickness in maize

The DNA of the two parents and the BC_4_F_4_ populations was extracted using the cetyltrimethylammonium bromide method [23], and genotyping of the DNA was performed using the maize 10 K liquid chip (MolBreeding, Shijiazhuang, China). A total of 10,910 SNP markers were obtained after excluding markers with heterozygosity greater than 0.1, biased segregation ratio greater than 0.3, and minimum allele frequency (MAF) less than 5%. 2,834 high quality SNPs were used for further research. The parameter recombination frequency of QTL IciMapping (version 4.1) [24] was set to be less than 0.35, and the Kosambi mapping function was used for the calculation of genetic map distances, and QTL for pericarp thickness were analyzed using inclusive composite interval mapping. The genetic distance information was imported into R4.2.1 (R Core Team (2023). R: A Language and Environment for Statistical Computing. R Foundation for Statistical Computing, Vienna, Austria. https://www.R-project.org/.) and plotted as a 2D genetic map using LinkageMapView’s “lmv.linkage.plot” command [25]. QTL nomenclature was in the form of “q + abbreviation of trait name + chromosome number” [26].

### Functional structural domain prediction and DNA sequencing analysis of candidate genes

Prediction of functional structural domains using the MaizeGDB database (B73 RefGen_V4) [27] and InterPro website [28]. The primers of candidate genes for PCR amplification were designed using NCBI primer-BLAST [29] and synthesized by Tsingke Biotech Co., Ltd (Tsingke, Guangzhou, China). Table S1 provides the primer sequences for the candidate genes. The PCR reaction volume was 25.0 μl containing 200 ng of genomic DNA, 1 μl of primer (10 μM) and 10 μl of 2 × plus Taq HiFi PCR mix (Mikx, Guangzhou, China). The quality of the amplification products was assessed by electrophoresis on a 1.2% agarose gel. Subsequently, the PCR products meeting the criteria were forwarded to Tsingke Biotech Co., Ltd. (Tsingke, Guangzhou, China) for sequencing. Sequence comparisons and analysis were performed using SnapGene software (www.snapgene.com). Schematic representation of gene structure and protein functional structural domains done by IBS software [30].

### Quantitative real-time PCR

RNA from the seedling leaf of B73 and Baimaya was extracted with RNA extraction reagent (Surbiopure, Guangzhou, China) and reverse transcribed into cDNA with All-in-one First-Strand Synthesis MasterMix with dsDNase (Xinkailai, Guangzhou, China). The cDNA was diluted 5 times and used as the template for quantitative real-time PCR (qPCR). *DUF* was used as a housekeeping gene [31]. qPCR was performed using CFX Connect™ Real-Time PCR Detection System (Bio-Rad, USA) and ChamQ SYBR qPCR Master Mix without ROX (Vazyme, Guangzhou, China). Quantification of qPCR results using the 2^−△△CT^ method [32]. Primer sequences for related genes are listed in Table S2. Three biological replicates were carried out.

## Results

### Dynamic changes in pericarp thickness in sweet, waxy, and normal maize during kernel development

**Fig. 1:**
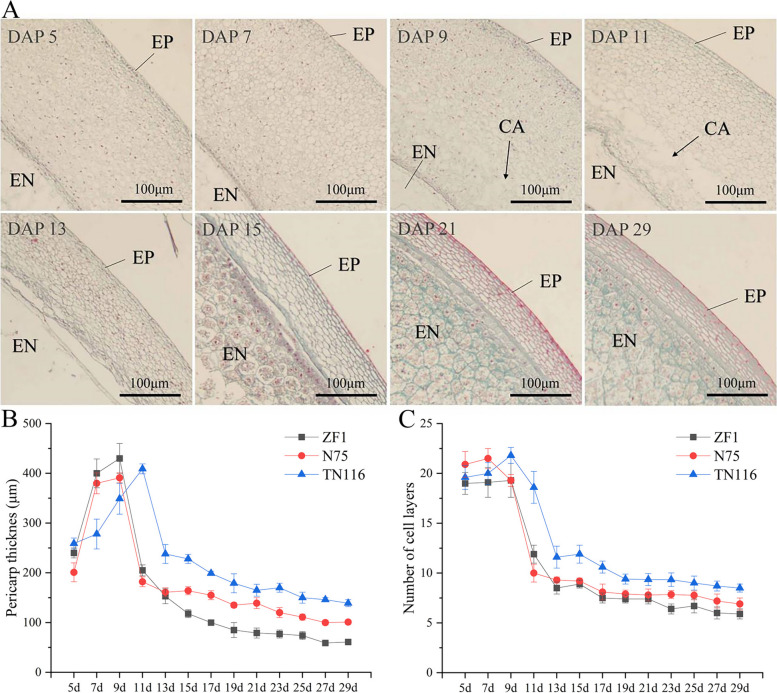
Changes in maize pericarp thickness during the kernel developmental process. **A** Microscopic observation of the pericarp development in maize. EP: exocarp. EN: endosperm. CA: the cavities formed by apoptotic cells. **B** Variation in pericarp thickness after self-pollination of three different corn types. **C** Variation in the number of cell layers after self-pollination. ZF1: sweet corn inbred line; N75: normal corn inbred line; TN113: waxy corn inbred line

There were significant differences in the thickness of the pericarp of sweet, waxy, and normal maize, but the trends in thickness showed consistency, with all showing an increasing and then gradually decreasing trend. Throughout the entire process of maize pericarp development, the number of mesocarp cells is the highest, and their morphological changes are the most dramatic. They constitute the major component that influences pericarp thickness, typically consisting of 5 to 20 cell layers (Fig. 1). In the initial stages following fertilization, pericarp thickness rapidly increases, which can be divided into two parts based on cell morphology: cell division and cell elongation. Before DAP 5, the increase in pericarp thickness is mainly due to the continuous division of mesocarp cells, resulting in a rapid increase in cell number. From DAP 5 to DAP 9, the number of cell layers stabilizes between 19–22 layers. Meanwhile, the cells start elongating both horizontally and vertically. During this stage, the increase in pericarp thickness is primarily caused by cell expansion (Fig. S1). After reaching its peak, there is a sharp decrease in pericarp thickness, which is also a result of rapid changes in the number of mesocarp cells. From DAP 11 to DAP 13, mesocarp cells undergo autophagy from the inside out, causing a significant disappearance of cells and the formation of cavities in the pericarp. The number of cell layers in the pericarp decreases from the initial 19–22 layers to 9–11 layers. As the cell number decreases and the substances in the cavities are absorbed by the endosperm, pericarp thickness rapidly decreases. In the subsequent period, changes in mesocarp cells tend to stabilize, and pericarp thickness no longer exhibits drastic changes but shows a gradual downward trend.

**Fig. 2:**
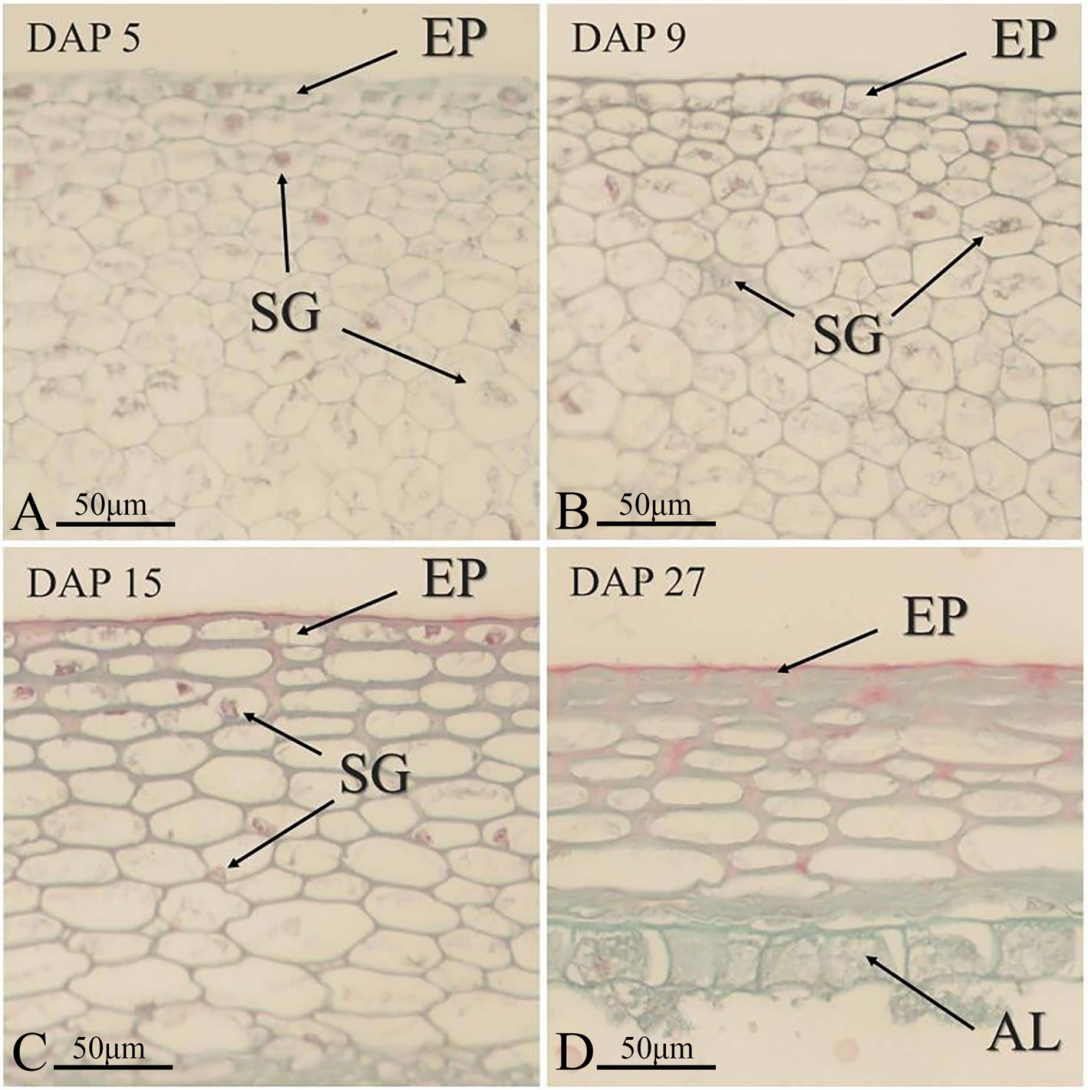
Changes in maize pericarp cells during various developmental stages. EP: exocarp. SG: starch grain. AL: aleurone layer

Studying the changes in the developmental process of the maize pericarp revealed that the process can be divided into four stages: growth, formation, autophagy, and maturation (Figs. 1 and 2). (1) Growth stage: From the time of self-pollination till DAP 5, the number of dividing pericarp cells, mainly mesocarp cells, rapidly increases. At the same time, these newly generated pericarp cells begin to synthesize starch. (2) Formation stage: from DAP 5 to DAP 9, the pericarp cells stop dividing and start to elongate. At the same time, large amounts of starch are present in the pericarp cells. The primary role of the pericarp cell at this stage is to synthesize and store starch. (3) Autophagy stage: from DAP 10 to 26, the pericarp cells stop synthesizing starch. Autophagy starts to occur in the mesocarp cells, the cells break down, and the contents flow out. This creates a cavity in the pericarp, and the starch stored in the cells is gradually absorbed by the endosperm. (4) Maturation stage: After DAP 27, the nucleus and contents of the pericarp cells mostly disappear, indicating that the pericarp cells have died. The major function of the pericarp at this stage is to protect the embryo and endosperm.

In summary, this study revealed that the differences in pericarp thickness among the three selected maize inbred lines and the differences in pericarp thickness at different developmental stages of individual kernels are mainly determined by the individual pericarp cell thickness and number of mesocarp cells.

### Phenotypic and genetic heritability analyses of pericarp thickness in maize

**Fig. 3:**
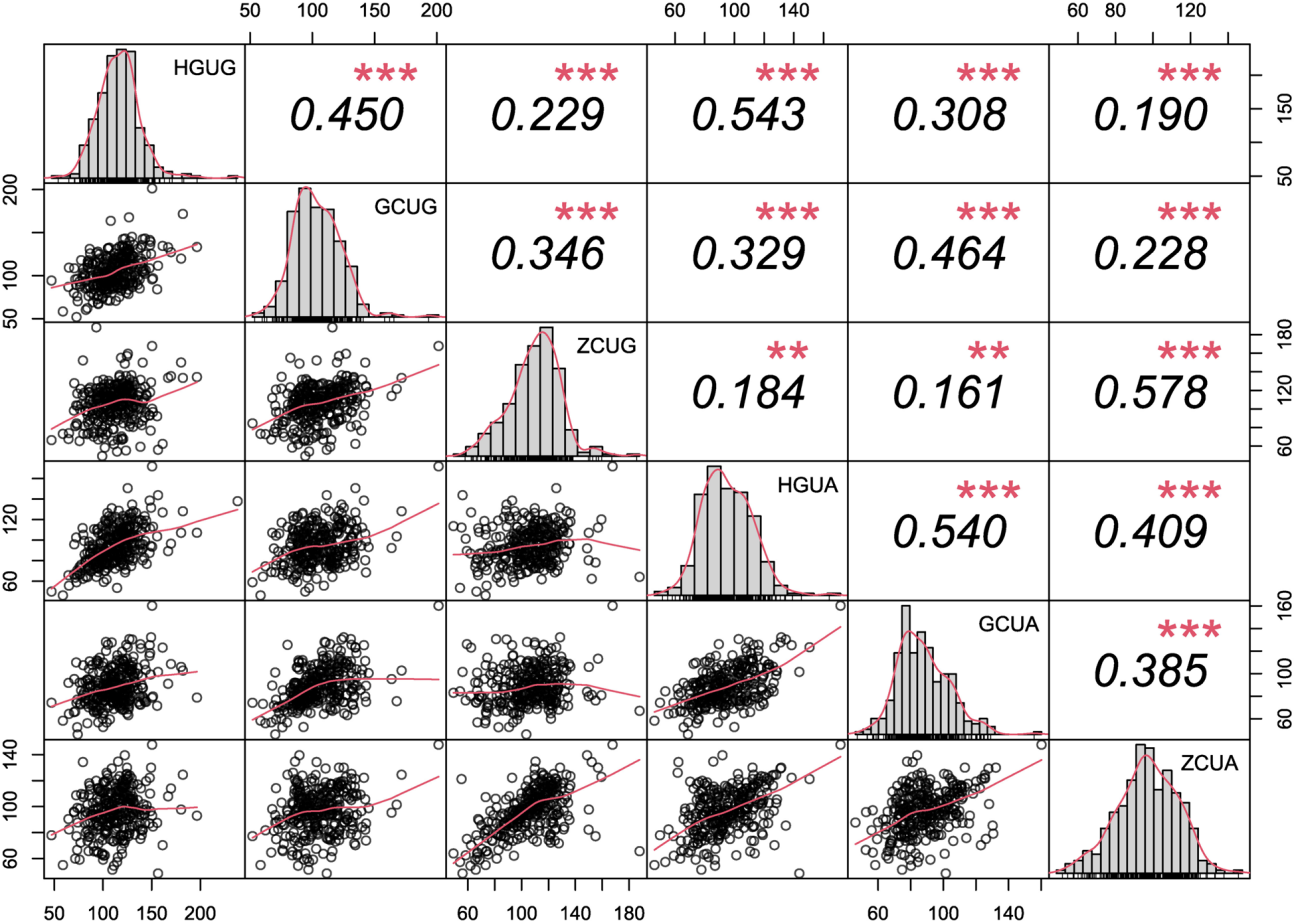
Pearson correlation coefficients for pericarp thickness in three different environments. The diagonal line represents the frequency histogram of peel thickness. The lower left of the diagonal (lower triangle) is a scatter plot of the expression values of the two samples. The greater the slope of the red fitted curve, the stronger the correlation between the two samples. In the upper right of the diagonal (upper triangle), the numbers indicate the correlation values between the two samples and * indicates the level of significance (* *P* < 0.05, ** *P* < 0.01, *** *P* < 0.001). HG: Huanggang, GC: Gucheng, ZC: Zengcheng, UG: The thickness of upper germinal side of pericarp, UA: The thickness of upper abgerminal side of pericarp

The phenotypic correlations of pericarp thickness in three different environments were positive and highly significant (Fig. 3). The correlation coefficients for the thickness of upper germinal side of pericarp (UG) and the thickness of upper abgerminal side of pericarp (UA) were 0.235–0.415 and 0.354–0.508, respectively. UG and UA (Fig. S2) was significantly correlated (*P* < 0.01) in the three different environments, indicating that both UG and UA can reflect the overall level of pericarp thickness in each individual line.

**Fig. 4:**
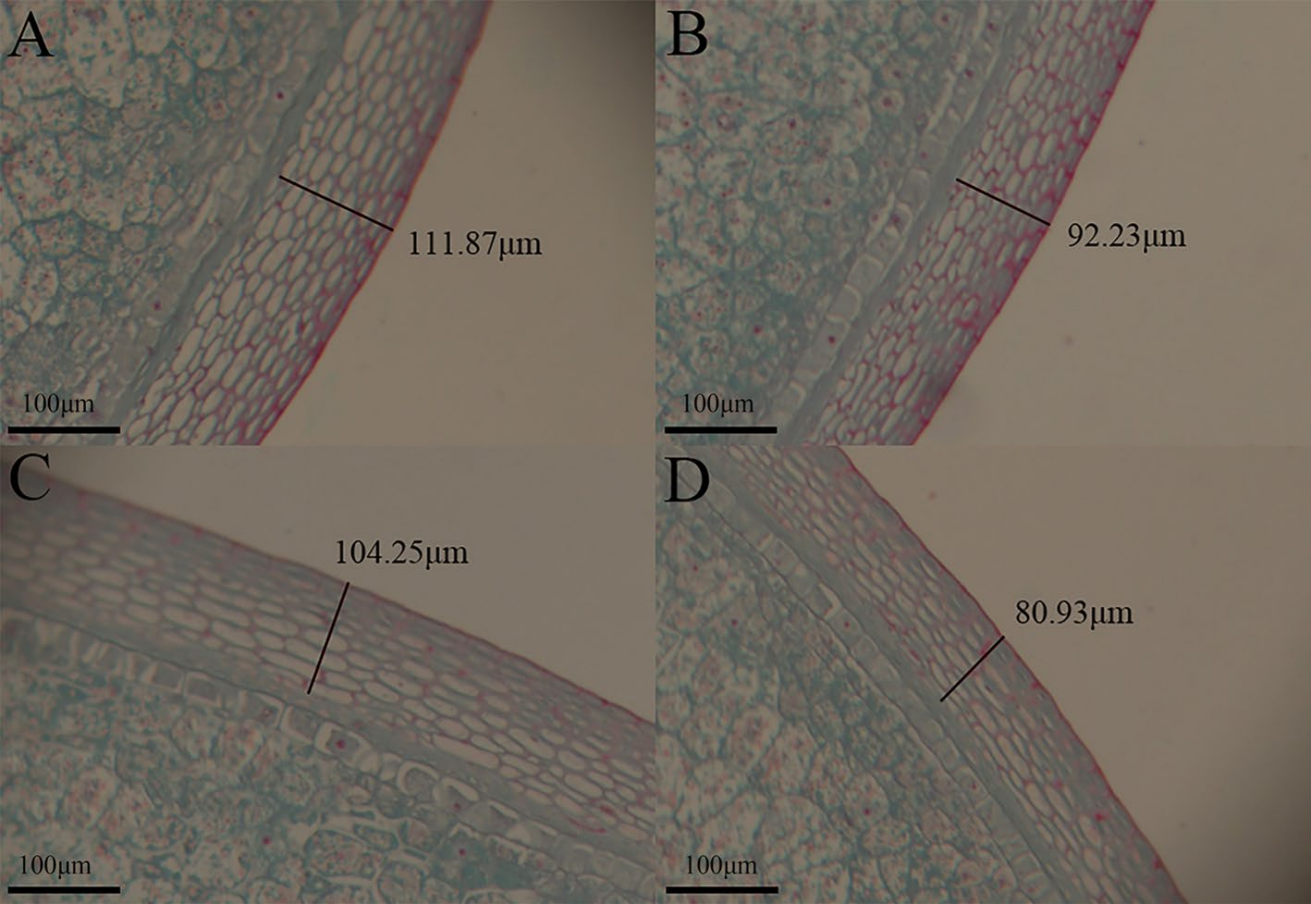
Sectioning of B73 and Baimaya to determine pericarp thickness. The thickness of the upper germinal side of the pericarp of (**A**) B73 and (**B**) Baimaya. The thickness of the upper abgerminal side of the pericarp of (**C**) B73 and (**D**) Baimaya

**Fig. 5:**
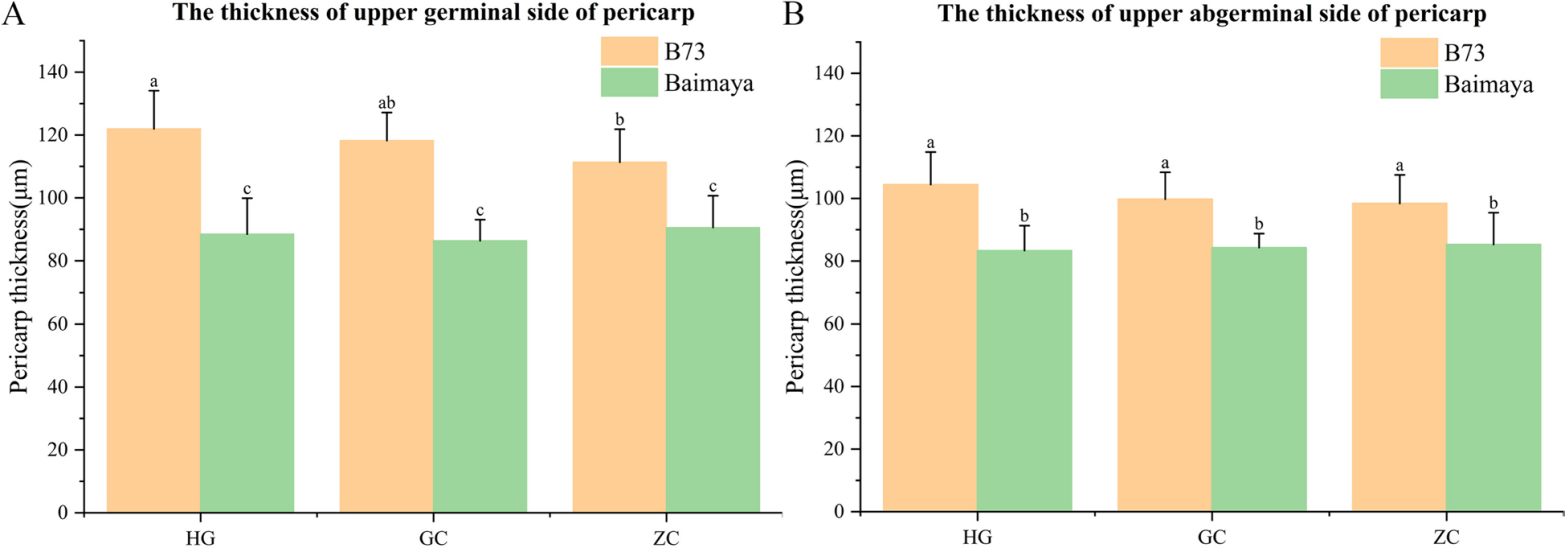
Analysis of significant differences in pericarp thickness between B73 and Baimaya. **A** The thickness of upper germinal side of pericarp. **B** The thickness of upper germinal side of pericarp. Different letters indicate significant differences at the *P* < 0.05 level

There was a significant difference in pericarp thickness between the two parents, with the pericarp of the paternal Baimaya being thinner than that of the maternal B73 (Figs. 4 and 5). The main reason for this difference was the different thickness of individual pericarp cells (Fig. S3). The thickness of the UG was slightly more than that of the UA, which was consistent across the three environments.

The broad-sense heritability (*h*) of the UG and UA were 0.63 and 0.70, respectively (Table 1). The segregation of pericarp thickness conformed to a normal distribution (Fig. S4) and was a typical quantitative trait under polygenic control. The pericarp thickness distribution in the BC_4_F_4_ population was continuous, and considerable transgressive segregation was observed, indicating that both parents contributed to alleles for pericarp thickness.

**Table 1:** Pericarp thickness in the two parents and BC_4_F_4_ population

Trait	Pericarp thickness in the parents (mean ± SD)		Pericarp thickness in BC_4_F_4_ population (mean ± SD)					
	B73 ± SD^a^	Baimaya ± SD^a^	Range^a^	Mean ± SD^a^	Skewness	Kurtosis	CV	$h_b^2$
HGUG	121.95 ± 12.14	88.46 ± 11.45	47.16–238.34	112.37 ± 22.43	0.80	3.50	21.56%	0.63
GCUG	118.25 ± 8.90	86.35 ± 6.72	51.93–201.45	105.28 ± 19.86	0.64	1.73	20.67%	
ZCUG	111.32 ± 10.52	90.56 ± 10.15	49.35–187.78	105.54 ± 20.41	− 0.03	0.85	20.97%	
HGUA	104.4 ± 10.41	83.31 ± 8.00	49.20–198.37	94.74 ± 18.41	0.70	2.27	21.36%	0.70
GCUA	99.8 ± 8.61	84.23 ± 4.54	46.10–160.25	88.76 ± 16.43	0.56	0.81	20.21%	
ZCUA	98.4 ± 9.13	85.23 ± 10.21	48.47–147.70	96.38 ± 17.13	− 0.17	− 0.07	19.52%	

*HG* Huanggang, *GC* Gucheng, *ZC* Zengcheng, *UG* the thickness of upper germinal side of pericarp, *UA* the thickness of upper abgerminal side of pericarp, *SD* standard deviation, *CV* coefficient of variation, $h_b^2$ broad-sense heritability

^a^ μm

### Identification of primary QTLs for pericarp thickness in the BC_4_F_4_ family lines

A genetic map was constructed using 2,824 polymorphic SNP markers (Fig. S5). There were 10 linkage groups in the map with a total distance of 4506.87 centimorgans (cM), with the longest linkage group being 749.56 cM and the shortest being 292.17 cM with an average distance of 1.57 cM. The maximum number of SNP markers was 414 in linkage group 1, and the minimum number was 199 in linkage group 7. The proportion of marker gaps of < 5 cM was calculated for each cluster to measure the degree of linkage between markers, with higher proportions indicating a more even distribution of markers in that cluster (Table S3). The highest proportion of gap of < 5 cM was 99.64% for linkage group 9, and the lowest was 93.89% for linkage group 2. Additionally, the largest gap of this map was located on linkage group 2 at 39.82 cM.

The QTL mapping for pericarp thickness in maize was based on the phenotypic data of the two replicates and three locations. The results of correlation analysis of pericarp thickness revealed that UG was significantly correlated with that of UA (Fig. 3). This indicated that the pericarp thickness of the two sides might be controlled by different QTLs, therefore, the data of the two sides were analyzed using QTL mapping. A total of 17 QTLs for pericarp thickness were detected in the BC_4_F_4_ population, distributed on maize chromosomes 1, 2, 4, 5, 8, 9, and 10 (Table 2).

**Table 2:** QTLs detected for pericarp thickness in the BC_4_F_4_ population

Trait	QTL name	Chr^a^	Physical Position (bp)^b^	QTL region (cM)	LOD	PVE (%)	ADD
HGUG	*qPT1-1*	1	212,215,145–212,948,882	437.5–440.5	7.96	4.93	− 11.54
	*qPT5-1*	5	200,168,546–200,881,691	55.5–57.5	2.74	3.01	4.25
	*qPT5-2*	5	25,824,467–31,694,189	248.5–250.5	5.92	5.68	4.27
	*qPT5-3*	5	15,976,063–25,824,467	251.5–252.5	5.75	3.78	2.81
GCUG	*qPT1-1*	1	212,215,145–212,948,882	437.5–440.5	10.46	10.69	− 11.61
	*qPT2-1*	2	2,550,197–14,732,993	358.5–362.5	3.40	3.75	− 10.82
	*qPT9-1*	9	2,212,304–2,340,596	63.5–67.5	6.40	8.23	2.54
	*qPT10-1*	10	23,813,974–25,984,314	226.5–227.5	3.82	3.75	− 2.96
ZCUG	*qPT1-1*	1	212,215,145–212,948,882	437.5–440.5	8.57	6.82	− 12.08
	*qPT4-1*	4	56,995,558–167,223,879	32.5– 37.5	3.06	3.61	3.07
	*qPT8-1*	8	156,015,875–161,738,256	2.5–5.5	3.40	2.80	15.56
	*qPT8-2*	8	140,913,186–142,387,066	33.5–38.5	2.76	2.15	− 11.41
HGUA	*qPT2-1*	2	2,550,197–14,732,993	358.5–364.5	4.06	6.31	− 11.03
	*qPT2-2*	2	139,801–845,641	369.5–373.5	4.25	5.88	4.07
	*qPT4-3*	4	181,253,653–181,460,287	520.5–525.5	3.85	5.24	− 4.26
GCUA	*qPT2-1*	2	2,550,197–14,732,993	359.5–364.5	8.20	8.63	− 13.13
	*qPT4-2*	4	168,862,323–169,541,635	242.5–248.5	2.61	2.00	4.34
	*qPT9-2*	9	97,782,073–100,421,417	167.5–171.5	4.46	3.83	6.56
	*qPT9-3*	9	136,271,947–137,747,972	351.5–354.5	5.56	5.53	− 0.22
ZCUA	*qPT2-1*	2	2,550,197–14,732,993	358.5–364.5	4.03	6.33	− 10.61
	*qPT5-4*	5	208,399,071–209,737,085	25.5–30.5	2.99	3.92	5.18
	*qPT9-4*	9	136,271,947–136,334,050	351.5–355.5	2.68	4.24	1.06

*LOD* Logarithm of odds

*PVE* phenotypic variance explained

^a^ Chr: chromosome

^b^ Physical positions of the identified QTLs are based on the reference sequence of B73 (B73 RefGen_V4 genome; https://www.maizegdb.org/)

A total of 10 QTLs for UG were identified, and the phenotypic variation explained by a single QTL ranged from 2.15% to 10.69%. *qPT1-1*, *qPT2-1*, *qPT10-1*, and *qPT8-2* exhibited negative additive effects. This indicated that the alleles of these QTLs that reduce pericarp thickness originated from the B73 parent, whereas the alleles carried by the Baimaya parent acted to reduce pericarp thickness in the QTLs with positive additive effects. Among them, *qPT8-1* exhibited the highest additive effect of 15.56. *qPT1-1* was identified in three different environments. This QTL is located at the physical position 212,215,145–212,948,882 on chromosome 1, explained 4.93%–10.69% of the phenotypic variance, and had high LOD values ranging from 7.96 to 10.46.

**Fig. 6:**
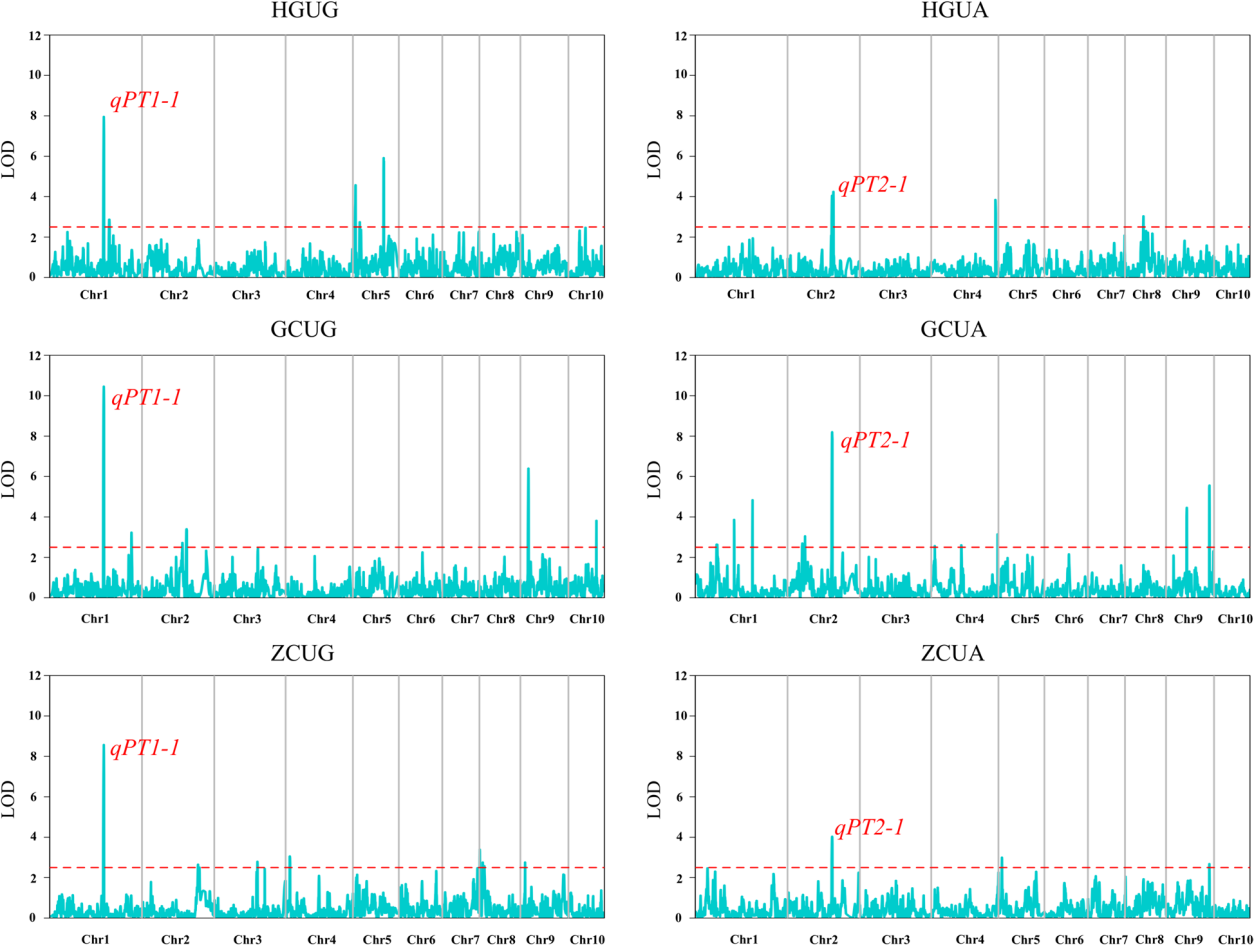
QTL mapping of maize pericarp thickness. The horizontal dotted line in the figure is the threshold line for a LOD value of 2.5, and the reddish labelled font is the major QTL

For UA, eight QTLs were identified. *qPT2-1* identified at Gucheng exhibited the highest LOD value of 8.20. The additive effect was − 13.13, representing that the allele for this QTL that reduces pericarp thickness originated from the B73 parent. Furthermore, *qPT2-1* was identified in both UG and UA. It is speculated that the pericarp thickness on both sides of the maize kernel may be controlled by this QTL. In summary, *qPT1-1* and *qPT2-1* were speculated to be stable QTLs controlling maize pericarp thickness (Fig. 6, Table 2).

### DNA sequencing of candidate genes and prediction of functional structural domains

The B73 reference sequence (B73 RefGen_V4) from the Maize Reference Genome Database was used to obtain genetic and functional annotation information within QTL intervals. In total, 14 and 662 genes were found within *qPT1-1* and *qPT2-1*, respectively (Tables S4 and S5). Through functional annotation and DNA sequence analysis, candidate genes (*Zm00001d001964* and *Zm00001d002283*) potentially controlling pericarp thickness were identified.

**Fig. 7:**
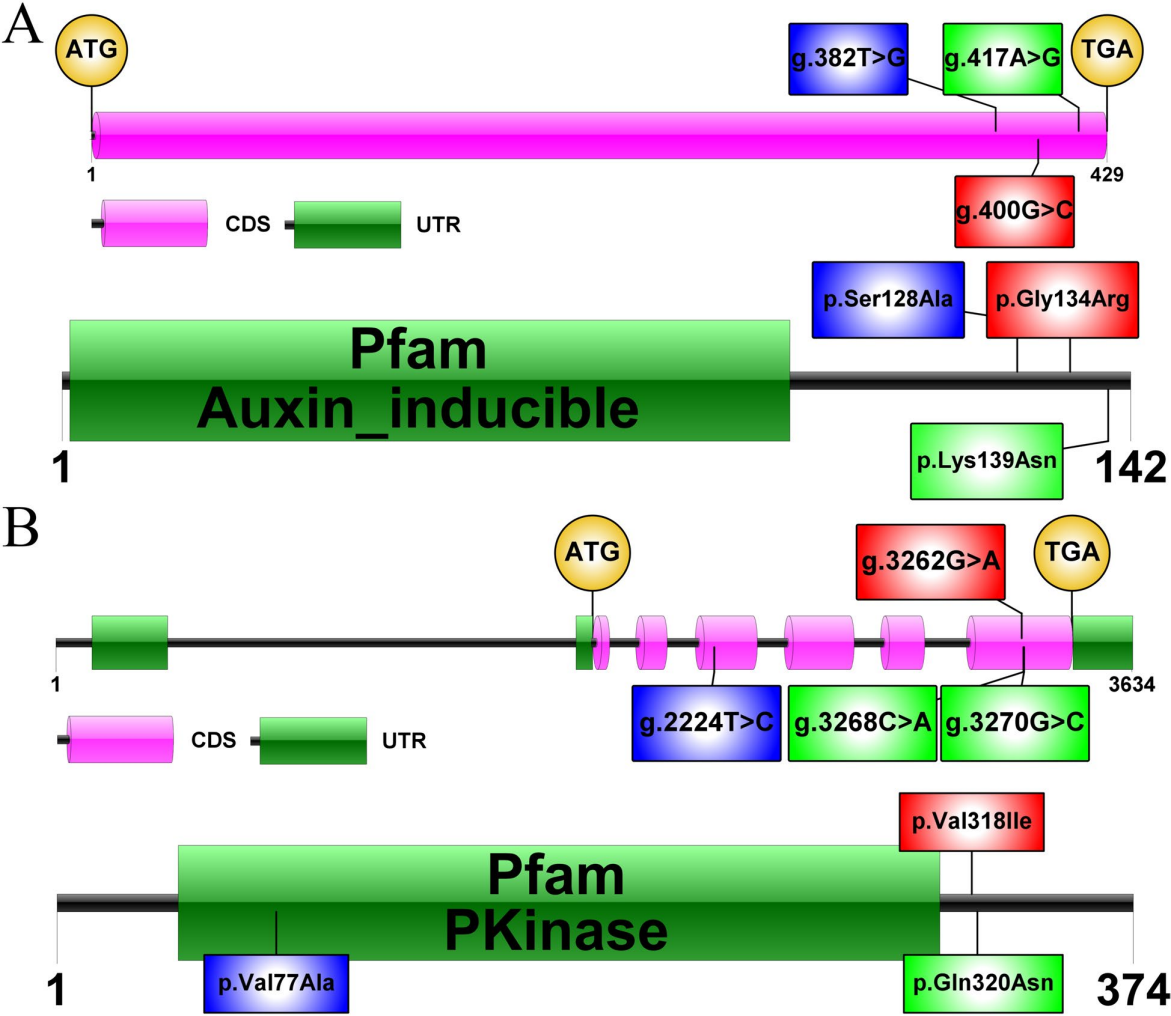
Schematic gene structure and protein functional structure domains (**A**) *Zm00001d001964 *(**B**) *Zm00001d002283*

Through the prediction of functional domains, Zm00001d001964 (ZmSAUR15) has an Auxin_inducible domain (Fig. 7A). It is well known that auxin influences cell division and cell elongation, thereby affecting plant growth and development, and consequently the thickness of the maize pericarp. Many members of the *SMALL AUXIN UP RNA* (*SAUR*) gene family are among the most quickly and significantly triggered by auxin [33]. The results of the DNA sequencing analysis indicate that the *Zm00001d001964* gene of Baimaya differs from B73 by three base substitutions, located at positions 382 bp, 400 bp, and 417 bp within the coding sequence (CDS). These substitutions result in the conversion of Serine to Alanine, Glycine to Arginine, and Lysine to Asparagine.

Furthermore, the analysis of functional domains revealed that Zm00001d002283 has a PKinase domain (Fig. 7B). Protein phosphorylation plays a vital role in regulating cell cycle progression, especially through the activity of cyclin-dependent protein kinases, which are key players in controlling cell division [34]. The DNA sequencing analysis of the *Zm00001d002283* gene reveals eleven base substitutions, two deletions, and one insertion in Baimaya compared to B73 (Table S6). Among these changes, four base substitutions were identified within the CDS at positions 2224 bp, 3262 bp, 3268 bp, and 3270 bp. These substitutions cause Valine to change to Alanine, Valine to Isoleucine, and Glutamine to Asparagine. The change from Valine to Alanine occurs within the PKinase domain. The sequencing peaks of some SNPs are shown in Fig. S6.

In addition, the results of qPCR showed that the gene expression of *Zm00001d001964* was higher in B73 than that in Baimaya, while the gene expression of *Zm00001d002283* was higher in Baimaya (Fig. S7). This suggests that high expression of *Zm00001d001964* promotes pericarp thickness, while high expression of *Zm00001d002283* reduces pericarp thickness.

## Discussion

### Cytological changes in the pericarp of different varieties of maize during kernel development

Generally, four methods are used for determining the thickness of maize pericarp: (1) Microscopic method in which sections of frozen seeds are stained with a dye and observed under a calibrated microscope [35]. (2) Weighing the whole pericarp and reporting it as a percentage of the total dry kernel weight [36].(3) Micrometer method, in which the pericarp is first peeled from the kernels and dried at room temperature, and finally, the thickness is measured using a micrometer [37]. (4) Method using penetrometer (an instrument used to assess the hardness of the fruit), which quantifies the thickness of the pericarp by calculating the ratio of spring pressure to surface area [38]. Microscopic method was used in this study. The pericarp thickness of different maize varieties exhibited significant variation. At DAP 27, reaching a minimum of 59 μm in sweet maize and a maximum of 139 μm in waxy maize. Notably, there was an observable trend of initial increase followed by decrease in pericarp thickness across different maize varieties. The trend of pericarp thickness was similar to that of Zhang et al. [9]. Furthermore, by analyzing the changes in pericarp thickness and disparities in pericarp cell structure, maize pericarp development has been classified into four stages: growth, formation, autophagy, and maturation. These findings are consistent with the research on other grass species, such as wheat [39].

During growth and formation, the thickness of the pericarp gradually increases. The main reason for the thickening of the pericarp during the growth stage is the dramatic increase in cell number due to the continuous division of the pericarp cells. The main physiological activity of the pericarp cells during the formation stage changes from cell division to cell elongation, and the main reason for the continued increase in pericarp thickness at this time is the change in the thickness of individual cells. Large amounts of starch are commonly accumulated in the pericarp cells of cereals [40–42]. In this study, large number of starch granules were observed to be synthesized and stored in the pericarp cells during the formative stages of maize pericarp development. From the autophagy stage onward, maize pericarp thickness continued to decrease and eventually stabilized. Programmed cell death (PCD) occurs in pericarp cells during cereal development and endosperm development is also affected by this process [43, 44]. During the autophagy stage of maize pericarp development, mesocarp cells enter into the PCD process. A large number of cells begin to break down, and the contents flow out of the pericarp to form a cavity. Further, the starch and other nutrients in the cavity are transported to the endosperm and stored there. This phenomenon is an important reason for the sharp decrease in the thickness of the pericarp. The maturation stage is the final stage of maize pericarp development. After the autophagy stage, most mesocarp cells die, and the pericarp thickness is close to the minimum value. At this time, almost all the starch in the pericarp cells is transported to the endosperm. With the dehydration of the kernel, the water content of the pericarp cells also gradually decreases, and the overall thickness of the pericarp remains unchanged.

### Comparisons of QTLs controlling pericarp thickness in maize

Previous studies have demonstrated a significant negative correlation between pericarp thickness and pericarp tenderness, and that pericarp thickness is controlled by multiple genes [3, 15]. Table 1 shows that the broad-sense heritability of the UG and UA were 0.63 and 0.70, respectively. It indicated that genetic effects had a significant influence on this trait, and environmental effects had little effect on pericarp thickness.

Yu et al. observed eight QTL associated with pericarp thickness on chromosomes 2, 3, 5, 6 and 8 using a BC_1_F_2_ population containing 190 families. Among them, the QTLs located in bin2.01, bin5.06 and bin8.05 were in similar regions to *qPT2-1*, *qPT5-1* and *qPT8-2* in this study, respectively [45]. In addition, a study involving 264 F_2:3_ family lines generated from crosses between Korean glutinous maize inbred lines BH 20 and BH 30 detected a total of 33 QTLs for pericarp thickness. These QTLs were found to be located on chromosomes 1, 2, 3, 4, 8, 9, and 10. The marker intervals umc1278 to umc1833 (bin:1.07), umc1329 to dupssr34 (bin:4.06), and umc1691 to umc1771 (bin:9.03) are in similar regions to *qPT1-1*, *qPT4-2*, and *qPT9-2*, respectively [16]. Using pericarp weight as a reference for measuring pericarp thickness, Wanlayaporn et al. identified QTLs associated with maize pericarp thickness on chromosomes 1, 2, 4, 9 and 10 [36]. Wu et al. conducted a study where they developed a BC_4_F_3_ population comprising 148 families. They constructed a genetic map with 3876 specific length amplified fragment tags and identified 14 QTLs associated with pericarp thickness. Among these QTLs, *qPT10-5* (located at position 144,631,242–145,532,401) was identified as the major locus. Within the *qPT10-5* region, they found 42 candidate genes. Through transcriptome analysis, they identified five genes that exhibited differential expression between the two parents. Based on gene annotation information, three of these genes were identified as potential candidate genes for pericarp thickness. Specifically, GRMZM2G143352 was annotated as an AUX/IAA transcription factor, GRMZM2G143402 as a ZIM transcription factor, and GRMZM2G143389 as a protein named FATTY ACID EXPORT 2 chloroplastic [46].

In this study, the pericarp thickness of the upper abgerminal and upper germinal was used as a representative to determine the maize pericarp thickness. A total of 17 QTL were finally identified by combining the phenotypic data on maize pericarp thickness with the genotypic data obtained from the maize 10 K SNP microarray. Two main effect QTLs were identified on chromosomes 1 and 2, respectively, with *qPT1-1* explaining 4.93%–12.97% of the phenotypic variance and *qPT2-1* explaining 4.04%–8.63% of the phenotypic variance. Furthermore, *qPT4-2*, *qPT5-1*, *qPT8-2*, and *qPT9-2* were consistent with the QTL results in other articles, although these loci were not repeatedly detected in this study.

### Candidate genes for maize pericarp thickness

In maize, the expression of *Zm00001d001964* is influenced by the presence of auxin. Auxin, a vital plant hormone that regulates the growth and development of plants, can induce the expression of auxin-responsive genes, including *AUX/IAAs*, *GH3s*, and *SAURs* [33]. The down-regulation of *Sl-IAA17*, which acts as an active inhibitor of gene transcription regulated by auxin, results in an increase in tomato pericarp cell size [47]. *SAUR19* has been found to have a pivotal role in facilitating cell elongation in Arabidopsis. This effect is achieved by stimulating the activity of the plasma membrane H-ATPase while suppressing the phosphatase type 2C-D subfamily [48]. In addition to *SAUR19*, there are other *SAURs* in Arabidopsis that play a positive role in cell expansion, such as *SAUR36*, *SAUR41*, and *SAUR63* [49–51]. Li et al. overexpressed the *VvSAUR041* gene from tomato and found that the average cell area of the pericarp tissue was greater in the OE lines [52]. Functional structural domain prediction of Zm00001d001964 revealed an Auxin-inducible structural domain. In contrast to the B73 reference gene, Baimaya exhibits three base substitutions, all occurring within the coding sequence. Auxin regulates cell division and expansion, thus controlling plant growth and development, which may impact maize pericarp thickness. These findings suggest that *ZmSAUR15* likely plays a crucial role in regulating pericarp thickness development.

The homologue of *Zm00001d002283* in Arabidopsis is *AT1G16670*, and its encoded product belongs to the protein kinase family. Protein kinases play a role in regulating the phosphorylation state of proteins and affect protein activity through catalysis of substrate proteins, which in turn affects a wide range of plant activities [53]. Protein phosphorylation regulates various biological processes such as signal transduction, growth and development, substance metabolism, and environmental responses by altering the conformation of proteins and affecting their activity and protein–protein interactions [54, 55]. Sequencing analysis of *Zm00001d002283* showed that Baimaya had eleven base substitutions, two deletions, and one insertion when compared to the reference gene for B73. Four of these base substitutions were in the CDS. Functional structural domain prediction revealed that Zm00001d002283 has a PKinase domain, and there is a change from Valine to Alanine within the PKinase domain. Protein kinases have a variety of important functions in living organisms. It can regulate the function and activity of proteins through phosphorylation reactions. In living organisms, protein kinases are involved in important biological processes such as cell signaling, cell cycle regulation, cell differentiation and proliferation. Czerednik et al. found that cyclin-dependent kinase (CDK) affect the size and number of cell layers in tomato pericarp cells [56]. A previous study found that overexpression of a zinc finger gene, *Solanum lycopersicum PERICARP-ASSOCIATED ZINC FINGER PROTEIN 1* (*SlPZF1*), enhanced the expression of cyclin-dependent protein kinase (*SlCDKB1* and *SlCDKB2*) in the pericarp tissue and reduces pericarp cell thickness [57].

In summary, analysis of the dynamics of pericarp thickness showed that changes in thickness at different developmental stages of the pericarp were mainly attributed to expansion and cell division of mesocarp cells. Our sequencing results indicate that the *Zm00001d001964* and *Zm00001d002283* genes of Baimaya show multiple base substitutions at the CDS compared to the B73 reference genome. These substitutions result in amino acid changes, which are associated with specific protein functional domains. In tomato, overexpression of the *VvSAUR041* gene increases the average cell area of the pericarp tissue and plays a positive role in fruit development [52]. Whereas, enhanced expression of cell cycle protein-dependent protein kinase in pericarp tissues decreased pericarp thickness and mesocarp cell size [57]. These are consistent with the qPCR results of this experiment (Fig. S7).

## Conclusions

This study investigated changes in maize pericarp development and identified major QTLs for maize pericarp thickness. During the development of maize kernel, pericarp thickness mainly exhibits a pattern of first increasing and then decreasing. By observing the changes in the structural characteristics of pericarp cells, it was found that the differences in pericarp thickness of individual kernels at different developmental stages were mainly determined by the thickness of individual pericarp cells and the number of mesocarp cells, and that pericarp development was divided into four stages: growth, formation, autophagy, and maturation. The pericarp thickness rapidly increases during the growth stage and reaches the maximum during the formation stage. Further, it begins to decrease during the autophagy stage and stabilizes during the maturation stage. Additionally, a high-density genetic map with a total length of 4,467.48 cM and 10 linkage groups was constructed using the BC_4_F_4_ population in combination with a maize 10 K SNP microarray, and the average genetic distance between adjacent markers was 1.57 cM. A total of 17 QTLs related to pericarp thickness were identified by combining phenotypic data and genetic mapping. The results revealed that *qPT1-1* was the main QTL controlling UG and explaining 4.93%–12.97% of phenotypic variation, whereas *qPT2-1* was the main QTL controlling UA and explaining 4.04%–8.63% of phenotypic variation. In addition, two candidate genes, *Zm00001d001964* and *Zm00001d002283*, were screened for possible control of maize pericarp thickness, in combination with functional annotation, DNA sequencing and qPCR analysis. Among them, Zm00001d001964 has an Auxin_inducible domain, while Zm00001d002283 has a PKinase domain. The application of these candidate genes to the breeding process is expected to help improve maize pericarp thickness and provide valuable guidance and insights to breeders. However, further studies and experiments are needed to confirm the functions and effects of these genes.

### Supplementary Information

**Supplementary Material 1. **

**Supplementary Material 2. **

## Data Availability

All data generated or analyzed during this study are included in this article and its supplementary information files or are available from the corresponding author on reasonable request.
