# Supplementary material for: Genetic analysis and QTL mapping for pericarp thickness in maize (Zea mays L.)
Source: BMC Plant Biol. 2024 Apr 25;24:338. doi: 10.1186/s12870-024-05052-1 (PMC11044598; doi:10.1186/s12870-024-05052-1)
Supplement: Supplementary file 1 — Supplementary Material 1. [file 12870_2024_5052_MOESM1_ESM.docx]

**Fig. S1.** Significance analysis of (A) pericarp cell thickness and (B) number of pericarp cell layers from DAP 5 to DAP 9. ZF1: sweet corn inbred line; N75: normal corn inbred line; TN113: waxy corn inbred line. Different letters indicate significant differences at the P<0.05 level.


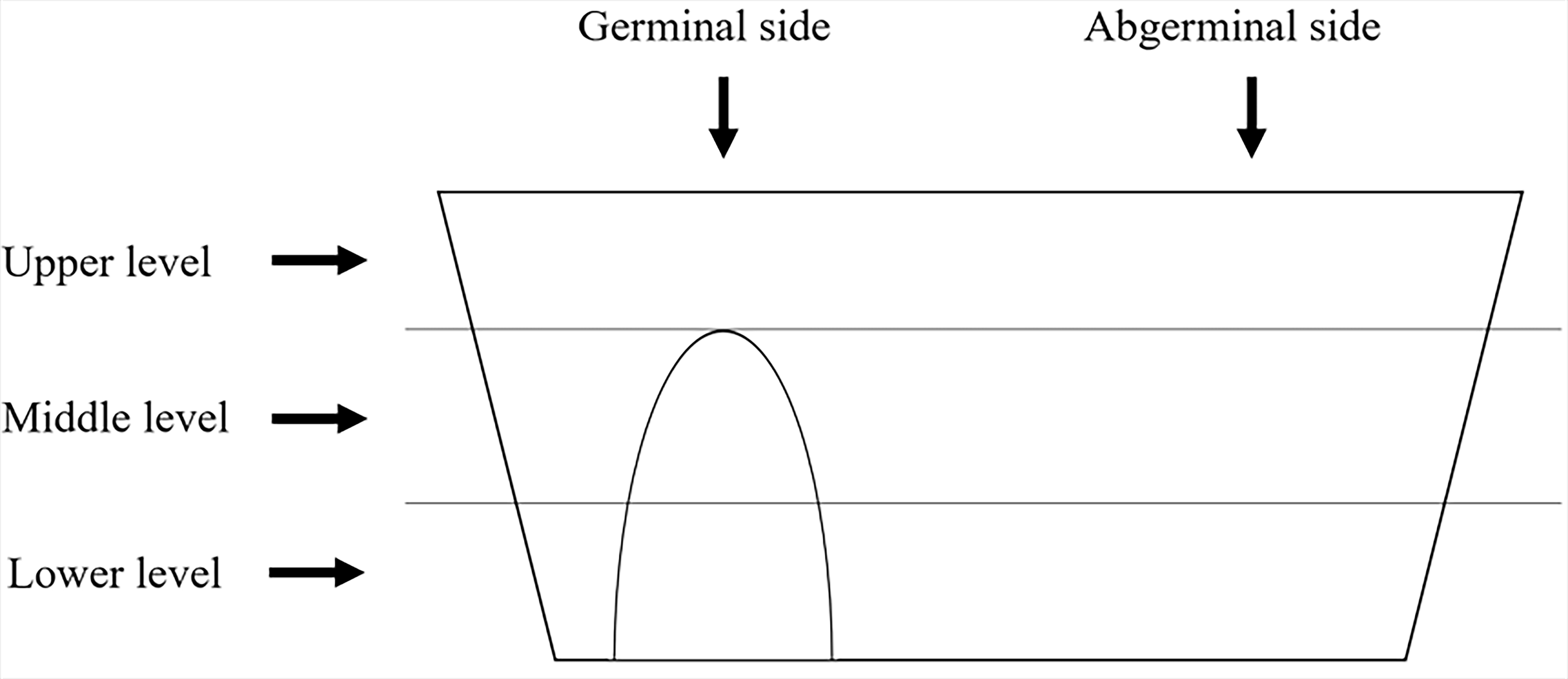


**Fig. S2.** Location of pericarp measurements





**Fig. S3.** Analysis of significant differences in the number of pericarp cell layers and pericarp cell size. (A, C) Upper germinal side of pericarp. (B, D) Upper germinal side of pericarp. Different letters indicate significant differences at the P<0.05 level.





**Fig. S4.** P-P plot of traits related to pericarp thickness in the BC_4_F_4_ family line.


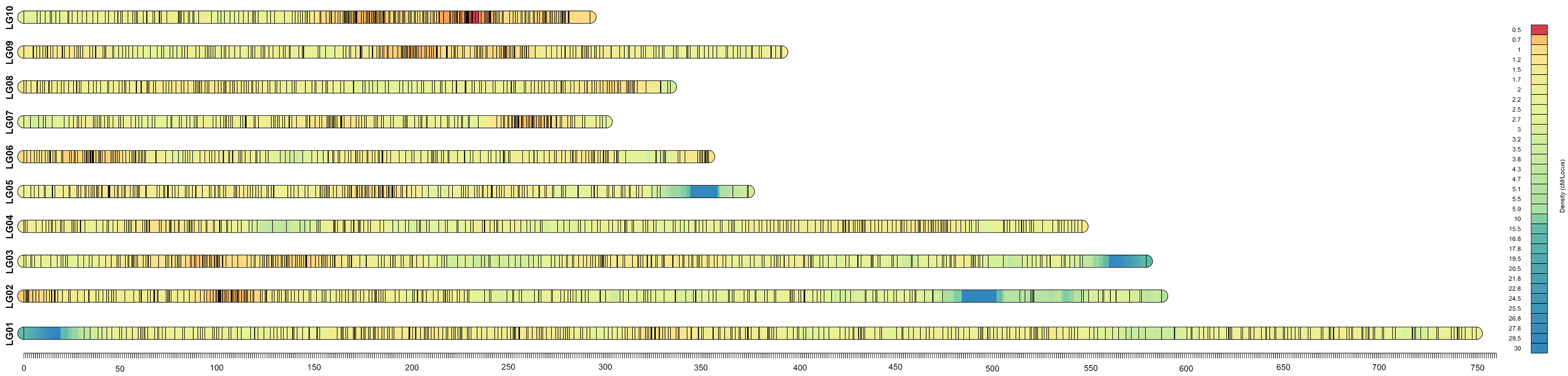


**Fig. S5.** High-density genetic linkage mapping of the BC_4_F_4_ population. The left side is the linkage group number, the lower scale is the genetic distance, and the right side is the marker density.


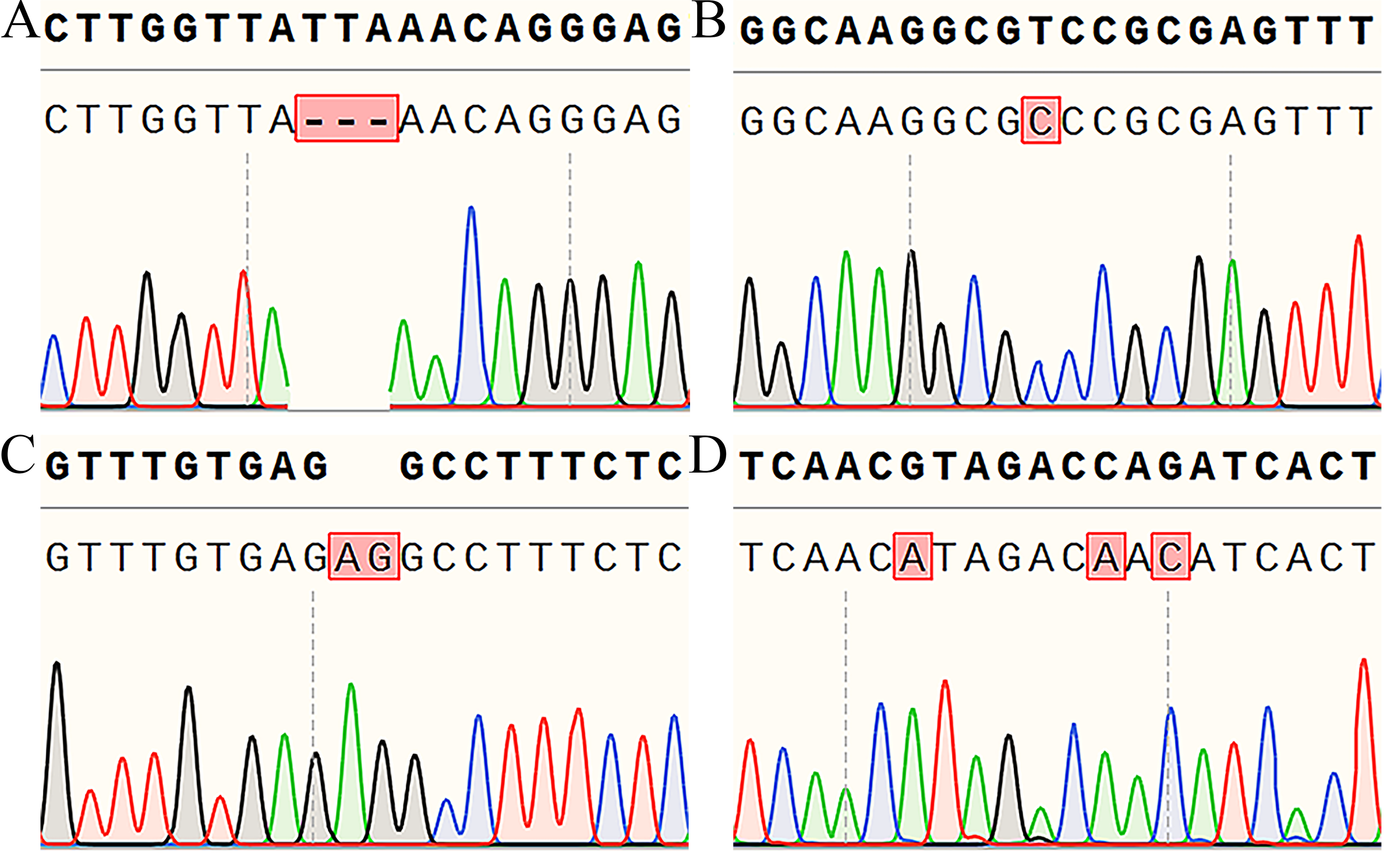


**Fig. S6.** Peak chart of the SNPs of *Zm00001d002283* in Baimaya. (A) g.2152_2154delTTA

(B) g.2224T>C (C) g.2730_2731insAG (D) g.3262G>A, g.3268C>A, g.3270G>C





Fig. S7. Significance analysis of relative gene expression of two candidate genes in B73 and Baimaya. (A) *Zm00001d001964* (B) *Zm00001d002283*. Different letters indicate significant differences at the P<0.05 level.
